# Prevalence of parvovirus B19 infection in patients with hematological diseases in the Western Brazilian Amazon

**DOI:** 10.1016/j.htct.2026.106477

**Published:** 2026-05-30

**Authors:** Paulo Henrique Rodrigues de Souza, Anderson Nogueira Barbosa, Leonardo Calheiros de Oliveira, Luma Silva Mineiro, Rosangela Santos de Abreu, Carlos Eduardo de Castro Alves, Matilde del Carmen Contreras Mejía, Felipe Gomes Naveca, Gemilson Soares Pontes

**Affiliations:** a Programa de Pós-Graduação em Hematologia, Universidade do Estado do Amazonas, Manaus 69065-001, AM, Brazil; b Laboratório de Virologia e Imunologia, Instituto Nacional de Pesquisas da Amazônia, Manaus 69060-001, AM, Brazil; c Fundação Oswaldo Cruz - Instituto Leônidas e Maria Deane, Manaus 69057-070, AM, Brazil

**Keywords:** Hematological diseases, Parvovirus B19V, Epidemiology, Prevalence, Blood center, Western Amazon

## Abstract

**Introduction:**

Parvovirus B19 (B19V) infects erythroid precursor cells, compromising red blood cell production. This can trigger severe anemia and other hematological diseases in susceptible patients. However, data on B19V epidemiology in patients with hematological diseases are scarce, especially in the northern region of Brazil. The aim of this study was to estimate the prevalence of IgG and IgM antibodies against B19V, and to detect B19 DNA, in patients with hematological diseases undergoing

**Methods:**

Plasma samples from 421 patients were subjected to enzyme immunoassay for the detection of anti-B19V IgG and IgM antibodies. Subsequently, all samples underwent real-time polymerase reaction for viral DNA detection.

**Results:**

More than half (55.11%) of the study population was seropositive for IgG anti-B19 antibodies. Notably, women (prevalence ratio: 1.62; 95% CI: 1.10–2.41; p-value = 0.016) and individuals aged 60 and above (prevalence ratio: 7.72; 95% CI: 3.77–15.80; p-value = 0.016) showed the highest seroprevalence rates. Patients with thrombophilia, lymphoma, and anemia also exhibited elevated seroprevalence, with thrombophilia having the highest rate (prevalence ratio: 6.52; 95% CI: 1.78–23.88). Twelve patients were positive for IgM anti-B19, and the presence of B19V DNA was confirmed in two patients (0.46%).

**Conclusions:**

Patients with hematological diseases, particularly thrombophilia, exhibit a high seroprevalence of B19V, indicating frequent prior exposure. However, recent infections and viremia are uncommon. The elevated seroprevalence among women and adults aged 60 and older suggests that these groups may be at greater risk. This underscores the need for enhanced epidemiological surveillance and targeted B19V testing to improve patient management.

## Introduction

Parvovirus B19 (B19V), discovered in 1975, belongs to the *Parvoviridae* family and the genus *Erythroparvovirus* [1]. Its genome is composed of single-stranded DNA (ssDNA) of approximately 5 kb that encodes three main proteins, VP1, VP2, and NS1, alongside several smaller accessory proteins that contribute to viral function [2]. It is classified into three genotypes (1, 2 and 3) and has an affinity for proerythroblasts in the bone marrow, binding to the P (Globoside) antigen on their membranes [3,4]. The NS1 protein has been identified as the main mediator of apoptosis in B19V-infected cells, playing a crucial role in the development of clinical complications [5]. This virus is commonly related as the causative agent of erythema infectiosum, popularly known as the "fifth disease", due to its distinctive skin rash [6].

B19V is ubiquitous, infecting most people at some point in their lives [7]. Its transmission occurs mainly through respiratory droplets, viremic blood transfusions, and the transplacental route [8]. Although B19V infections often manifest asymptomatically or with mild symptoms, it is notable that the virus has the ability to temporarily interfere with erythropoiesis, culminating in hematologic disorders [9]. Notable clinical manifestations include idiopathic thrombocytopenic purpura (ITP) and severe anemia in immunocompromised patients, alongside other disorders characterized by the suppression of erythropoiesis (red blood cell production) [10].

Some rare hematological manifestations have been associated with B19V infection since the identification of this virus and its tropism by proerythroblasts, including bicytopenias, pancytopenia, myelodysplastic syndrome and leukoerythroblastopenia [11]. Studies indicate a case frequency of 15%-30% of B19V infection in patients with acute lymphoblastic leukemia (ALL) [12,13]. Furthermore, studies conducted in Tunisia reported a prevalence of 65% for B19V infection among blood donors and 56.5% among patients with sickle cell disease [14,15].

A study conducted in Saudi Arabia investigated B19V infection in patients with sickle cell disease, revealing a prevalence of 37.6% in this population [16]. In the northern region of India, a prevalence of 53.4% of B19V infection was observed in children with hemato-oncological disorders [17]. In Brazil, the first reports of B19V infection date back to 1985, when three pregnant women were referred for the detection of rubella antibodies in Rio de Janeiro [18]. In addition, case studies point to B19V infection as a strong complicating factor in the clinical course of hematological diseases [18]. Despite its global prevalence in hematological disorders, comprehensive epidemiological studies on B19V, especially in Brazil's northern region, are lacking. Case studies underscore the significant impact of B19V on the clinical course of hematological diseases.

Individuals with hematologic disease infected with B19V have a decrease in the production of viable erythrocytes, exacerbating the disruption of hematologic homeostasis [19]. This decrease is particularly evident in patients with anemia and leukemia [20]. In patients with a compromised hematopoietic system, such as in cases of leukemia or bone marrow transplant recipients, B19V infection can lead to serious blood disorders, including pancytopenia, bone marrow necrosis syndrome, and fat embolism syndrome [21]. In some rare cases, B19V infection can also complicate the course of acute leukemia [13]. In patients with chronic hemolytic anemia, B19V infection can trigger an aplastic crisis, temporarily stopping the production of blood cells in the bone marrow and aggravating the anemia [22]. B19V-associated ITP is a condition that results in thrombocytopenia, characterized by a low platelet count in the blood. In children, ITP can develop as a chronic condition after an acute viral infection [23].

The intricate relationship between B19V infection and hematologic diseases underscores the pivotal role of epidemiological studies in shaping clinical strategies and public health interventions. By thoroughly estimating B19V prevalence across diverse hematological conditions, this knowledge not only enhances patient care but also guides preventive measures. Epidemiological studies serve as the initial step in developing tailored patient management approaches, facilitating a deeper understanding of the intricate link between viral infections and hematologic disorders. This study is a cross-sectional investigation of parvovirus B19V infection in patients with hematological diseases in the Western Brazilian Amazon.

## Materials and methods

### Ethical approval

This study was approved by Human Research Ethics Committee (CAAE 63474122.1.0000.0009) of the Foundation of Hematology and Hemotherapy of the Amazon state (HEMOAM). All individuals signed an informed consent form prior to their participation in the study. Consent for individuals under 18 years of age was obtained from one of the parents or legal guardians. Confidentiality and the right to withdraw from the study at any time were assured to all participants.

### Study design and population

This study was conducted from November 2022 to March 2023 at the HEMOAM Foundation (FHEMOAM) outpatient clinic and included patients with a range of hematological disorders. The study involved 421 patients with diverse hematological conditions residing in different cities and towns within the western Brazilian Amazon region. Both male and female patients with ages ranging from 1-96 years old were eligible for inclusion. Detailed demographic information was gathered from the electronic medical record system IDOCTOR at FHEMOAM. Employing a cross-sectional observational approach, the study adopted consecutive sampling as its selection method, with the sample size determined based on a 95% confidence interval.

### Serological analysis

Serum samples were tested for the presence of anti-B19V IgG and IgM antibodies using an enzyme immunoassay (Anti-Parvovirus B19 ELISA, Euroimmun, Lubeck, Germany), following the manufacturer's recommendations. The optical density (OD) resulting from the test was measured in a spectrophotometer (Molecular Devices, San Jose, CA, USA) employing a 450 nm filter. Positivity was estimated by calculating the ratio of the sample OD to the calibrator OD, considering the manufacturer's indicated cut-off values. Serum levels of anti-B19V IgG were estimated in IU/mL by interpolating the standard curve provided by the manufacturer. The assay demonstrates 100% sensitivity and specificity for anti-B19V IgG detection. For anti-B19V IgM, the sensitivity and specificity are 100% and 97.9%, respectively. The detection limit of the tests was defined as a value of three times the standard deviation of an analyte-free sample, with the final value being the lowest detectable antibody concentration. Seroprevalence was defined a priori by anti-parvovirus B19 IgG positivity, since IgG is the standard epidemiological marker used to assess previous infection and cumulative exposure within a population. Anti-B19V IgM positivity was analyzed separately as an indicator of recent infection and was not included in the main seroprevalence estimate.

### Nucleic acid extraction and B19V DNA detection

All serum samples underwent DNA extraction using the Blood Lysate protocol of the PureLink Genomic DNA Mini Kit (Invitrogen, Waltham, MA, USA), following the manufacturer's recommendations. Detection of B19V DNA was achieved by real-time polymerase reaction (PCR) on the QuantStudio 5 equipment (Applied Biosystems, Waltham, MA, USA). The amplification reaction utilized 200 ng of DNA, forward and reverse primers (5 µM; 5′-CAC CCC CAT GCC TTA TCA TC-3′ and 5′-TTG TAC GCT AAC TTG CCC AG-3′), and a FAM-labeled probe (10 µM; 5′-FAM-CAG TCA TGC AGA ACC TAG AGG A-3′). Procedures were conducted using the TaqMan Fast Virus 1-Step Master Mix (Applied Biosystems, Waltham, MA, USA) in a total volume of 10 µL. The reaction consisted in an initial denaturation at 95°C for 20 seconds, succeeded by 45 cycles consisting of 3 seconds at 95°C and 30 seconds at 60°C.

## Statistical analysis

Multinomial logistic regression and prevalence ratio (PR) were used to investigate the association between the likelihood of B19 infection and demographic variables or different types of hematological diseases. PR was determined by dividing the prevalence among individuals exposed to specific demographic characteristics or hematological diseases by the prevalence among those not exposed. Linear regression was used to test the relationships between plasma concentrations of anti-B19V IgG and continuous quantitative variables. The student's t-test and one-way ANOVA were used to test the difference between means of two or more variables, respectively. The results were considered statistically significant when the p-value was less than 0.05 for a 95% confidence interval (95% CI). R version 4.3.0 software was used for statistical analysis.

## Results

### Prevalence of B19V infection according to demographic factors and type hematological diseases

Because IgM primarily reflects recent infection, whereas IgG reflects previous exposure and may persist long term, IgM-positive results were not incorporated into the main estimate of seroprevalence, which was designed to represent cumulative prior exposure in the study population. The seroprevalence of B19V infection in the study population was 55.11% (95% CI: 0.50–0.60). Seroprevalence rates were higher in women than men (seroprevalence: 59.92% versus 40.14%; PR: 1.62; 95% CI: 1.10–2.41; p-value = 0.016). The seroprevalence increased with age, with the highest seroprevalence in patients over 60 years old (72.22%) and the lowest in young people aged 1–18 years (27.01%) (Table 1). Increasing age was positively correlated with a rise in seroprevalence (PR = 5.41 for the age group of 19–30 years compared to the reference group and PR = 7.72 for patients over 60 years old; p-value <0.0001 for both comparisons).

**Table 1:** Parvovirus B19 prevalence in the study population according to sex and age.

Category	n (%)	IgG positive n (%)	PR (95% CI)	p–value
Sex				
Men	169 (40.14)	81 (47.93)	Ref.	
Women	252 (59.86)	151 (59.92)	1.62 (1.10–2.41)	0.016
Age (years)				
01–18	137 (32.54)	37 (27.01)	Ref.	
19–30	66 (15.68)	44 (66.67)	5.41 (2.86–10.21)	<0.0001
31–40	48 (11.40)	33 (68.75)	5.95 (2.90–12.18)	<0.0001
41–50	63 (14.96)	44 (69.84)	5.81 (3.03–11.14)	<0.0001
51–60	53 (12.59)	35 (66.04)	5.26 (2.66–10.40)	<0.0001
60+	54 (12.83)	39 (72.22)	7.72 (3.77–15.80)	<0.0001

PR: Prevalence Ratio; 95% CI: 95% Confidence Interval; Ref.: Reference

The values of PR, 95% CI, and p-value were obtained from multinomial regression analysis

B19V seroprevalence varied depending on the type of hematological disease, with higher rates observed in patients with thrombophilia (88.89%), followed by lymphoma (61.40%), anemia (57.69%), and leukemia (43.40%). Seroprevalence rates were higher among the following patient groups: anemia (PR: 1.83; CI: 1.11–3.01; p-value = 0.018), lymphoma (PR: 2.08; 95% CI: 1.08–4.00; p-value = 0.029), chronic myeloid leukemia (PR: 5.24; 95% CI: 1.83–15.06; p-value = 0.002), and thrombophilia (PR: 6.52; 95% CI: 1.78–23.88; p-value = 0.005). Notably, patients with thrombophilia were 6.5 times more likely to have been exposed to B19V than those with other hematological diseases, such as leukemia. Conversely, patients with sickle cell anemia demonstrated a significantly lower likelihood of infection. (PR: 0.24; 95% CI: 0.08–0.67; p-value = 0.007) (Table 2).

**Table 2:** Parvovirus B19 seroprevalence according to hematologic disorders.

	Age range	Sex		Total (%)	IgG positive n (%)	PR (95% CI)	p–value
		F	M				
Leukemia	22 ± 30	55	51	106 (25.18)	46 (43.40)	Ref.	
ALL	11 ± 17	24	32	56 (52.83)	17 (30.36)	Ref.	
CLL	43 ± 81	3	2	5 (4.72)	2 (40.00)	1.53 (0.23–10.00)	0.657
AML	22 ± 39	10	8	18 (16.98)	8 (44.44)	1.84 (0.62–5.46)	0.275
CML	39 ± 52	15	8	23 (21.70)	16 (69.57)	5.24 (1.83–15.06)	0.002
Undetermined	01 ± 13	3	1	4 (3.77)	3 (75.00)	–	–
Anemia	34 ± 41	113	43	156 (37.05)	90 (57.69)	1.83 (1.11–3.01)	0.018
Iron-deficiency	37 ± 50	25	7	32 (20.51)	24 (75.00)	Ref.	
Aplastic	22 ± 49	8	7	15 (9.62)	13 (86.67)	2.17 (0.40–11.74)	0.37
Fanconi	26	1	–	1 (0.64)	–	–	–
Sickle Cell	15 ± 26	21	15	36 (23.08)	15 (41.67)	0.24 (0.08–0.67)	0.007
Hemolytic	26 ± 46	11	4	15 (9.62)	9 (60.00)	0.50 (0.14–1.85)	0.298
Megaloblastic	52 ± 72	14	2	16 (10.25)	8 (50.00)	0.43 (0.12–1.53)	0.191
Microcytic	13 ± 36	2	1	3 (1.92)	2 (66.67)	0.67 (0.05–8.36)	0.753
Pernicious	48	–	1	1 (0.64)	–	–	–
Undetermined	31 ± 46	31	6	37 (23.72)	19 (51.35)	–	–
Lymphoma	34 ± 41	25	32	57 (13.54)	35 (61.40)	2.08 (1.08–4.00)	0.029
Hodgkin's	20 ± 30	15	12	27 (47.37)	17 (62.96)	Ref.	
Non-Hodgkin's	42 ± 56	9	19	28 (49.12)	17 (60.71)	0.91 (0.31–2.70)	0.864
Undetermined	06 ± 28	1	1	2 (3.51)	1 (100.00)	–	–
Hemophilia	13 ± 25	2	19	21 (4.99)	13 (61.90)	2.12 (0.81–5.54)	0.125
Leukopenia	51	1	–	1 (0.24)	1 (100.00)	–	–
Myeloma	39 ± 65	4	4	8 (1.90)	3 (37.50)	0.78 (0.18–3.44)	0.746
Thrombocytopenia	32 ± 51	15	8	23 (5.46)	14 (60.87)	2.03 (0.81–5.10)	0.132
Polycythemia	51 ± 72	2	–	2 (0.48)	1 (50.00)	1.30 (0.08–21.42)	0.852
ITP	19 ± 36	16	7	23 (5.46)	8 (34.78)	0.70 (0.27–1.78)	0.449
Thrombophilia	47 ± 61	14	4	18 (4.27)	16 (88.89)	6.52 (1.78–23.88)	0.005
Von Willebrand	15 ± 56	5	1	6 (1.43)	5 (83.33)	6.52 (0.74–57.76)	0.092
Total		252	169	421	232(55.11)		

PR: Prevalence Ratio; 95% CI: 95% Confidence Interval; Ref.: Reference; ITP: Idiopathic Thrombocytopenic Purpura; ALL: Acute Lymphoblastic Leukemia; CLL: Chronic Lymphocytic Leukemia; AML: Acute Myeloid Leukemia; CML: Chronic Myeloid Leukemia; F: Female; M: male

The values of PR, 95% CI, and p-value were obtained from Multinomial Regression Analysis. Numbers in bold indicate statistically significant values

The prevalence of anti-B19V IgM was 2.85% (12/421), indicating recent serological evidence of infection. Most IgM-positive individuals were women (n = 9) and were aged 30 years or older (n = 8) (Table 3). Of the IgM-positive patients, seven (58.33%) were also positive for IgG anti-B19V. B19V DNA was detected in two patients (0.46%) and was analyzed separately as molecular evidence of detectable viremia. Notably, both patients were negative for anti-B19V IgG and IgM. One of the patients positive for B19V DNA had acute lymphoblastic leukemia, and the other had chronic myeloid leukemia. The overall positivity, defined as the presence of one or more laboratory markers (anti-B19V IgG, IgM, or DNA), was 56.77% (239/421). However, this composite measure was not used as the primary endpoint because these markers reflect different stages or manifestations of B19V infection.

**Table 3:** Positive cases for the presence of IgG and IgM anti-B19V in patients with hematological diseases.

Hematological diseases	Sex	Age	IgM	IgG
Leukemia (Undetermined)	F	1	+	–
ITP	F	10	+	–
Thrombocytopenia	M	10	+	–
Aplastic Anemia	F	12	+	+
Hodgkin's Lymphoma	F	30	+	+
Non-hodgkin's lymphoma	M	30	+	+
Thrombocytopenia	F	39	+	+
AML	M	50	+	+
Megaloblastic Anemia	F	69	+	–
Non-hodgkin's lymphoma	F	70	+	–
Thrombophilia	F	72	+	+
Anemia (Undetermined)	F	75	+	+

ITP: Idiopathic Thrombocytopenic Purpura; AML: Acute Myeloid Leukemia; F: Female; M: Male; +: Positive; –: Negative.

### Analysis of serum anti-B19V IgG levels

**Figure 1:**
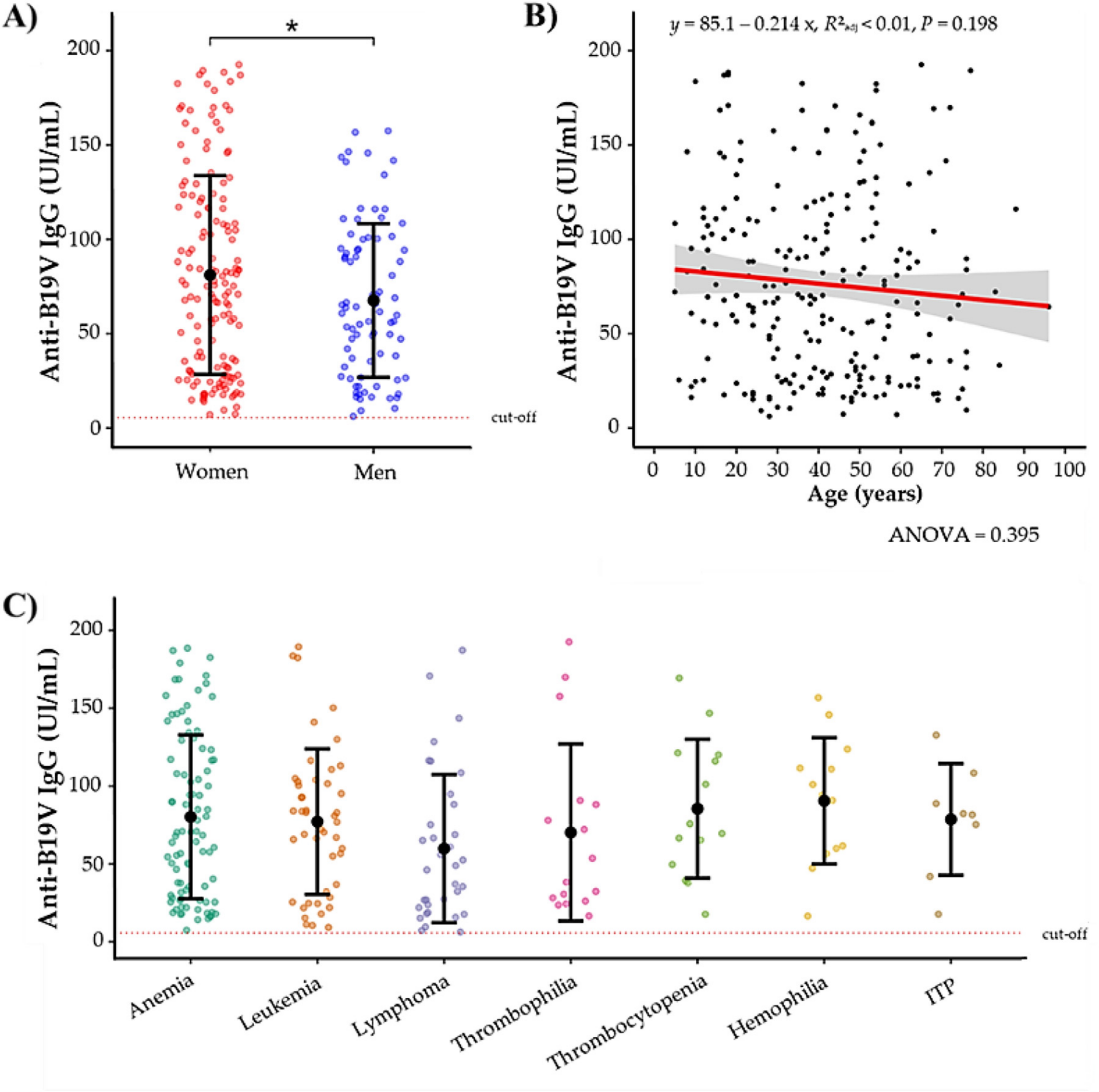
Serum anti-B19V IgG antibody levels in patients with hematological diseases. Serological analysis of serum anti-B19V IgG levels according to sex (A), age (B) and type of hematological disease (C). The difference between means of serum anti-B19V IgG antibody levels by sex was assessed using a student’s t-test. The correlation between serum anti-B19V IgG antibody levels and age was evaluated by linear regression. Data in A and B are represented by mean and standard deviation. *p-value = 0.046.

Serum anti-B19V IgG levels were higher in women than in men (81.11 ± 52.67 IU/mL; p-value = 0.046) (Figure 1A). There was no correlation between anti-B19V IgG serum levels and age (Figure 1B). While patients with hemophilia (90.48 ± 40.63 IU/mL), thrombocytopenia (85.42 ± 44.59 IU/mL), and anemia (80.21 ± 52.62 IU/mL) exhibited numerically higher serum anti-B19V IgG levels, these differences were not statistically significant compared to other hematological conditions (Figure 1C).

## Discussion

The prevalence of B19V infection has been assessed in many countries, including China, the United States, Saudi Arabia, Turkey, and Croatia, with prevalence ranging from 20%-60% [16,24, 25, 26, 27]. However, few studies have addressed the circulation of B19V among patients with hematological diseases worldwide. In the context of Brazil, particularly in the western Brazilian Amazon region, there is limited epidemiological information available on B19V infection.

This study found a high seroprevalence of B19V infection (55.11%) in individuals with hematological diseases in the western Brazilian Amazon, which was slightly higher than that found in other regions of Brazil (30%-53%) and India (53.40%), but much higher than that found in Tanzania (29%), Saudi Arabia (37.6%), and the United States (35%) [16,17,27, 28, 29, 30, 31]. These findings underscore the importance of understanding regional variations in B19V prevalence and its potential impact on individuals with severe hematologic conditions.

In this study, women exhibited higher seroprevalence rates and notably elevated serum IgG anti-B19 levels compared to men. Freitas et al. found a higher seroprevalence of B19V infection among women (59.70%) than men (35.40%) in the northern Brazilian city of Belém do Pará [32]. Similar findings emerged from a study in England and Wales, which reported a 28.5% higher seroprevalence of anti-B19V IgG among women compared to men [33]. Additionally, certain studies have reported a higher seroprevalence of B19V infection in pregnant women, often linked with fetal mortality [34, 35, 36]. The reasons behind the higher B19 infection rate in women are still elusive. Moreover, this study did not investigate the possible connection between pregnancy and B19V.

The findings of the present research demonstrated a positive correlation between age and B19V seroprevalence. Patients older than 60 years showed the highest seroprevalence, while patients aged 1 to 18 years showed the lowest. However, serum anti-B19V IgG levels did not correlate significantly with age. This pattern aligns with a study from São Paulo State, where sickle cell anemia patients ≥40 years had the highest B19V seroprevalence (93.3%) and those aged 1–9 years the lowest (20.7%), consistent with cumulative, age-related exposure. [31]. Another study conducted in Australia with healthy individuals also linked higher seroprevalence to B19V infection with increasing age, in which individuals aged 65 to 80 years showed the highest seroprevalence of B19V infection [7].

In Germany, the seroprevalence of B19V is around 20% in children aged 1 to 3 years and 67% in adolescents [37,38]. Adolescents may contribute to the spread of B19V infection in school settings, given the high rates of contact and transmission [37,38]. However, the seroprevalence of B19V infection in children and adolescents in this study was very low compared to older individuals. One plausible explanation for the distinct epidemiological pattern identified in Brazil might be that children and adolescents with hematological diseases, due to their fragile health, frequently miss school, where B19V transmission commonly occurs. However, this possibility was not specifically investigated.

In this study, patients with thrombophilia, lymphoma, anemia, and chronic myeloid leukemia had a high prevalence of B19V infection, suggesting an increased likelihood of past B19V exposure. However, no correlation was observed between serum anti-B19V IgG levels and the type of hematological disease. Susceptibility to B19V infection has been correlated with different hematological diseases, especially leukemia, lymphoma, and anemia [39]. Prior studies have demonstrated an increased susceptibility to B19 infection among patients with anemia, as evidenced by a significantly higher prevalence of B19V infection (87%) compared to those with other hematological malignancies, such as chronic myeloid leukemia (18.70%) and lymphomas (52%) [40,41]. Anemia is the main consequence of B19V infection due to the destruction of erythrocyte percussor cells during the viral cycle [42].

Strikingly, the findings of the present study revealed that patients with thrombophilia exhibited the greatest seroprevalence rate for B19V infection, which was up to six times higher than that of patients with other hematological diseases. To date, there is no known direct association between thrombophilia and B19V infection, but viral infections are known to be linked to blood clotting disorders [43]. For example, B19V can infect endothelial cells, which are key regulators of coagulation, thereby activating them and initiating the coagulation cascade [43,44]. Further studies with larger sample sizes are needed to confirm the association between thrombophilia and increased exposure to B19V infection, as well as the impact of this infection on the development and prognosis of this hematological disease.

Conversely, sickle cell anemia showed a lower likelihood of contracting B19V infection in this study. This contradicts previous findings that reported a high seroprevalence of B19V among individuals with sickle cell disease [45,46]. These contrasting outcomes warrant further investigation into potential factors influencing the interplay between sickle cell anemia and B19V infection, such as variations in genetic predisposition and environmental factors. Understanding these discrepancies may provide deeper insights into the complex relationship, shedding light on novel mechanisms influencing viral interactions within specific genetic conditions.

This study found that 12 patients were seropositive for IgM and seven were seropositive for both IgG and IgM anti-B19V antibodies. This finding is consistent with the results of a similar study in India, which reported that 53.40% of patients with hematological diseases were positive for IgG anti-B19V and 6.70% were positive for IgM antibodies [17]. Interestingly, only two patients in this study were positive for B19V DNA and neither of them had detectable IgM or IgG anti-B19V antibodies. This suggests that B19V infection in these cases may have been in its early stages, as B19V viremia is often short-lived and can be difficult to detect, especially if blood samples are not collected during this period [5].

Given the pivotal role of transfusions, the lack of mandatory B19V screening in Brazil and most international jurisdictions represents a significant transfusion-safety gap [47,48]. This gap in screening could facilitate the transmission of B19V through blood or plasma, posing serious health risks to patients with hematological conditions. Complications such as anemia, prolonged red blood cell aplasia, fever, erythema, and even pancytopenia may arise [49,50]. While lacking longitudinal data on post-transfusion outcomes, this study still offers crucial information for blood safety. Uncovering the prevalence of this under-researched virus is key to future decisions about mandatory screening.

This study had limitations, including restricted access to some medical records, which precluded a detailed assessment of specific diagnoses. In many instances, this information was incomplete, compromising further analyses. Another problem was related to the parents' refusal to allow children to participate in the study at the time of data collection. However, we understand the situation due to the sensitivity and severity of some patients. This limitation may have affected the representativeness of the sample in the case of some hematological diseases, such as myeloma and polycythemia.

Despite the limitations mentioned above, this study provides an important contribution to B19V surveillance in the Brazilian Amazon region, particularly among patients with hematological diseases. It presents valuable pioneering epidemiological information on the dynamics of B19V infection in this specific population, raising questions about its clinical implications.

## Conclusions

Parvovirus B19 is highly prevalent among patients with hematological diseases undergoing treatment at a renowned blood center in Manaus, Amazonas, Brazil. The data suggest marked cumulative B19V exposure, disproportionately affecting women and older individuals. Prior B19V exposure was more frequent in thrombophilia, lymphoma, anemia, and chronic myeloid leukemia, with thrombophilia showing the greatest prevalence. These data indicate substantial cumulative exposure but low active viremia, supporting targeted B19V testing in compatible clinical scenarios and motivating larger, longitudinal studies in the region. Given the central role of transfusions and the absence of mandatory B19V screening in Brazil, these findings also highlight a potential transfusion-safety gap warranting periodic surveillance.

## Authors’ Contributions

Conceptualization, Pontes GS and Souza PHR; methodology, Souza PHR, Barbosa AN, Alves CEdC, Oliveira LC, Mineiro LS, Mejía MdCC and Naveca FG; research, Souza PHR, Barbosa AN, Alves CEdC, Oliveira LC, Abreu RSA and Mineiro LS; formal analysis, Souza PHR, Barbosa AN, Alves CEdC, Mejía MdCC, Naveca FG, Oliveira LC and Mineiro LS; elaboration - preparation of original draft, Pontes GS, Souza PHR and Barbosa AN; Writing - review & editing, Pontes GS, Souza PHR, Barbosa AN, Alves CEdC; supervision, Pontes GS; project management, Pontes GS; resources/acquisition of financing, Pontes GS. All authors have read and agreed to the published version of the manuscript.

## Humans Ethics and Consent to participate declarations

This study was performed in line with the principles of the Declaration of Helsinki. Ethical approval was granted by Human Research Ethics Committee (CAAE 63474122.1.0000.0009) of the Foundation of Hematology and Hemotherapy of the Amazon state (FHEMOAM). All individuals signed the informed consent form prior to their participation in the study.

## Funding statement

This research was funded by the Coordination for the Improvement of Higher Education Personnel - CAPES (Finance code PROCAD AMAZÔNIA 88881.200581/201801).

## Data availability statement

The authors confirm that the data supporting the findings of this study are available within the article or its supplementary materials.

## Conflicts of interest

The authors have no conflicts of interest to declare for this study.
